# Higher Estimated Net Endogenous Acid Production May Be Associated with Increased Prevalence of Nonalcoholic Fatty Liver Disease in Chinese Adults in Hong Kong

**DOI:** 10.1371/journal.pone.0122406

**Published:** 2015-04-23

**Authors:** Ruth Chan, Vincent Wai-Sun Wong, Winnie Chiu-Wing Chu, Grace Lai-Hung Wong, Liz Sin Li, Jason Leung, Angel Mei-Ling Chim, David Ka-Wai Yeung, Mandy Man-Mei Sea, Jean Woo, Francis Ka-Leung Chan, Henry Lik-Yuen Chan

**Affiliations:** 1 Department of Medicine and Therapeutics, The Chinese University of Hong Kong, Hong Kong, China; 2 Centre for Nutritional Studies, The Chinese University of Hong Kong, Hong Kong, China; 3 Institute of Digestive Disease, The Chinese University of Hong Kong, Hong Kong, China; 4 Department of Imaging and Interventional Radiology, The Chinese University of Hong Kong, Hong Kong, China; 5 Jockey Club Centre for Osteoporosis Care and Control, The Chinese University of Hong Kong, Hong Kong, China; 6 Department of Clinical Oncology, The Chinese University of Hong Kong, Hong Kong, China; Medical University Innsbruck, AUSTRIA

## Abstract

Nonalcoholic fatty liver disease (NAFLD) has been associated with reduced growth hormone levels and signaling. Such hormonal changes also occur in metabolic acidosis. Since mild metabolic acidosis can be diet induced, diet-induced acid load may constitute a nutritional factor with possible influence on NAFLD development. This study explored whether a higher diet-induced acid load is associated with an increased likelihood of NAFLD. Apparently healthy Chinese adults (330 male, 463 female) aged 19-72 years were recruited through population screening between 2008 and 2010 in a cross-sectional population-based study in Hong Kong. Estimated net endogenous acid production (NEAP) was calculated using Frassetto’s method and potential renal acid load (PRAL) was calculated using Remer’s method based on dietary data from a food frequency questionnaire. NAFLD was defined as intrahepatic triglyceride content at >5% by proton-magnetic resonance spectroscopy. Possible advanced fibrosis was defined as liver stiffness at >7.9 kPa by transient elastography. Multivariate logistic regression models were used to examine the association between each measure of dietary acid load and prevalent NAFLD or possible advanced fibrosis with adjustment for potential anthropometric and lifestyle factors. 220 subjects (27.7%) were diagnosed with NAFLD. Estimated NEAP was positively associated with the likelihood of having NAFLD after adjustment for age, sex, body mass index, current drinker status and the presence of metabolic syndrome [OR (95% CI) = 1.25 (1.02-1.52), *p* = 0.022]. The association was slightly attenuated but remained significant when the model was further adjusted for other dietary variables. No association between PRAL and NAFLD prevalence was observed. Both estimated NEAP and PRAL were not associated with the presence of possible advance fibrosis. Our findings suggest that there may be a modest association between diet-induced acid load and NAFLD. More studies are needed to ascertain the link between diet-induced acid load and NAFLD and to investigate the underlying mechanisms.

## Introduction

Nonalcoholic fatty liver disease (NAFLD) is the most common cause of abnormal liver biochemistry worldwide. The prevalence of NAFLD is increasing in Asia. The prevalence of NAFLD across Asia varies from 5% to 40% [1, 2] NAFLD may progress to nonalcoholic steatohepatitis (NASH), cirrhosis, liver failure and liver cancer, and is believed to be the leading etiology for cryptogenic cirrhosis [3, 4]. NAFLD is also strongly associated with obesity and metabolic syndrome, and is shown to be an independent cardiovascular risk factor [5, 6]. Therefore, there is growing interest in determining risk factors for hepatic steatosis.

Hormonal changes have been linked to the development and severity of NAFLD. There is growing evidence to suggest that reduced levels of growth hormone (GH) and insulin-like growth factor-I (IGF-I) in adults is associated with an increased prevalence of NAFLD and progression to NASH or liver cirrhosis [7–9]. Meanwhile, substantial evidence from animal studies and human studies shows that metabolic acidosis suppresses GH secretion and IGF-I response [10, 11]. Diet composition can influence the acid-base balance [12]. Although diet-induced ‘low-grade’ metabolic acidosis has minimal adverse effects in short-term, a prolonged diet-induced high acid load may lead to a mild form of chronic metabolic acidosis and is considered to have significant clinical relevance [13]. Since NAFLD and metabolic acidosis are both associated with reduced levels of GH and IGF-I, it is possible to speculate that diet-induced acid load may constitute a nutritional factor with influence on NAFLD development.

To our knowledge, the association between diet-induced acid load and NAFLD risk has been rarely reported. Only one study has examined such association in a group of healthy Caucasian young men and women, and showed that higher dietary acidity in adolescence may be prospectively associated with hepatic lipid accumulation in females [14]. Although the study was prospective in nature, it had several methodological limitations including small sample size, selective sample, and use of alanine aminotransferase (ALT), hepatic steatosis index (HSI), and fatty liver index (FLI) as surrogates of NAFLD.

In view of the above limitations, the scarcity of evidence on this topic as well as the fact that Chinese diets are different from Western diets, the present study aimed to explore the association of dietary acid-base load with NAFLD prevalence in 793 Chinese aged 19–72 years, using data from a population-based study in Hong Kong. We speculated that higher dietary acid-base load was associated with an increased likelihood of NAFLD as measured using proton-magnetic resonance spectroscopy. Since NAFLD is benign in most of the cases, we also explored the association between dietary acid-base load and the likelihood of advanced fibrosis as measured using transient elastography.

## Materials and Methods

### Study population

This was a cross-sectional population-based study. The study population included 793 subjects who participated in a population screening for NAFLD. Details of the screening project have been reported previously [15, 16]. In brief, 1,069 subjects aged 18 to 72 years and randomly selected from the government census database agreed to participate in the screening project from May 2008 to September 2010. Those with active malignancy, metallic implants or other contraindications to magnetic resonance imaging, positive hepatitis B surface antigen or antibody against hepatitis C virus, secondary causes of fatty liver (e.g. consumption of amiodarone and tamoxifen) and decompensated liver disease (defined as bilirubin above 50 μmol/l, albumin below 35 g/l, platelet count below 150 × 10^9^/l, international normalized ratio above 1.3, or the presence of ascites or varices) (n = 147), with excessive alcohol use (defined as more than 20 g/d day in men and more than 10 g/d in women) (n = 30), with incomplete dietary data (n = 95), and without body mass index (BMI) data (n = 4) were excluded from the final analysis. The study protocol conformed to the ethical guidelines of the Declaration of Helsinki, and was approved by the Clinical Research Ethics Committee of The Chinese University of Hong Kong. All subjects provided informed written consent.

### Clinical assessment

At the baseline clinic visit, drug history, alcohol intake, smoking and past medical history were recorded using a standardized questionnaire. Alcohol consumption of 20 g daily (140 g weekly) for men and 10 g daily (70 g weekly) for women was the threshold to define NAFLD [17]. Anthropometric measurements including body weight, body height, waist and hip circumferences were obtained using standardized methods. BMI was calculated as weight (kg) divided by height (m) squared. Waist circumference (WC) was measured at a level midway between the lower rib margin and iliac crest with the tape all around the body in the horizontal position. Hip circumference (HC) was measured as the maximum circumference around the buttocks posteriorly and the symphysis pubis anteriorly. Waist-to-hip ratio (WHR) was calculated as the waist divided by the hip circumference.

Blood tests for liver biochemistry, glucose and lipids were taken after at least 8 hours of fasting. Metabolic syndrome was defined according to the ethnic-specific criteria by the International Diabetes Federation, which was modified from the National Cholesterol Education Program, Adult Treatment Panel III Guidelines, as any three of the followings: (i) waist circumference ≥90 cm in men and ≥80 cm in women; (ii) triglycerides >1.7 mmol/L; (iii) high-density lipoprotein-cholesterol <1.03 mmol/L in men and <1.29 mmol/L in women; (iv) blood pressure ≥130/85 mmHg; and (v) fasting plasma glucose ≥5.6 mmol/L; or receiving treatment for the above metabolic abnormalities [18].

### Proton-magnetic resonance spectroscopy (^1^H-MRS)

^1^H-MRS was performed to measure intrahepatic triglyceride content (IHTG) within 8 weeks from the baseline visit. Details of the scanning sequence and analysis have been described previously [15, 19]. Whole-body 3.0T scanner with a single voxel point-resolved spectroscopy sequence and an echo time of 40 ms and repetition time of 5,000 ms was used. Fatty liver was defined as an IHTG of 5% or more [20].

### Transient elastography

Liver stiffness measurement by transient elastography (Fibroscan, Echosens, Paris, France) was performed at the baseline clinic visit according to the instructions and training provided by the manufacturer. Ten successful acquisitions were performed on each subject. The success rate was calculated as the ratio of the number of successful acquisitions over the total number of acquisitions. The median value represented the liver elastic modulus. Liver stiffness measurements were considered reliable only if 10 successful acquisitions were obtained, the success rate was >60% and the interquartile range to median ratio of the 10 acquisitions was <0.3. Liver stiffness was expressed in kiloPascal (kPa). Operators must have performed at least 50 procedures before participating in this study, and were blinded to all clinical data and the diagnoses of the patients. Since the number of participants with advanced fibrosis was low (16 out of 654 available data), subsequent analyses used the presence of possible advanced fibrosis as an outcome instead. The cut-off value of 7.9 kPa was used to estimate the number of subjects with possible advanced fibrosis according to a local validation study using liver histology as the reference standard [21].

### Dietary assessment

Dietary intake was recorded using a modified version of a locally validated food-frequency questionnaire (FFQ) that captures food intake over 7 days [22] (S1 File). The questionnaire contained 297 food items in seven broad categories. To ensure data quality, the subjects filled in the questionnaires under the supervision of a trained research staff. Food replica, food containers, and a catalogue of pictures of individual food portions were provided to the subjects to assist portion size estimation. Daily nutrient intakes were estimated using Food Processor Nutrition analysis and Fitness software version 8.0 (ESHA Research, Salem, Oregon, USA) with the addition of local and Chinese food items [23–25]. All nutrient intakes were adjusted for dietary energy intake by the residual method for subsequent data analysis [26].

### Estimation of dietary acid load

Several algorithms have been developed to estimate acid load from diet [27]. Fassetto *et al*. calculated the estimated net endogenous acid production (NEAP) from the diet’s protein to potassium ratio [28] whereas Remer *et al*. estimated diet-induced potential renal acid load (PRAL) from average intestinal absorption rates of ingested protein and other minerals as well as an anthropometry-based estimate for organic acid excretion [29]. Each has its rationale and limitations [30]. In this study, both markers of diet-induced acid load were calculated. Frassetto’s method has been used in our previous study [31]. The method calculates the diet’s protein/potassium ratio expressed as g/mEq. The rationale and algorithm of this method have been described previously and the estimated NEAP could account for about 70% of the variation in renal net acid excretion [28]. We also performed additional analyses using the PRAL as a second marker for diet-induced acid load, and the PRAL was calculated using an algorithm described by Remer *et al*. [27]:

PRAL (mEq/d) = 0.4888 × protein intake (g/d) + 0.0366 × phosphorus (mg/d)–0.0205 × potassium (mg/d) – 0.0125 × calcium (mg/d) – 0.0263 × magnesium (mg/d)

Both the estimated NEAP and the PRAL were energy-adjusted using residual method for subsequent analyses [26].

### Statistical analysis

Statistical tests were performed using the Statistical Package for Social Sciences version 21.0 (SPSS Inc., Chicago, US). Histograms were used to screen for normal distribution. Logarithmic transformation was applied to skewed variables whenever appropriate. Continuous variables were expressed in mean ± SD and compared using the independent *t* test if they were normally distributed. Skewed variables were expressed in median (IQR) and compared using the Mann-Whitney U test. Categorical variables were compared using chi-squared test or Fisher exact test as appropriate. Pearson’s correlation or Spearman’s rank correlation was used to examine the correlation between nutrient intakes or food group intakes and estimated NEAP or PRAL whenever appropriate.

Binary logistic regression models were applied to assess both the unadjusted and the adjusted associations between estimated NEAP or PRAL and the presence of NAFLD. Variables with *p* <0.1 in the univariate analysis were entered into the multivariate analysis. The first multivariate model was adjusted for age, sex, BMI, being a current drinker, and the presence of metabolic syndrome. To avoid the multicollinearity problem, only the presence of metabolic syndrome was included in the multivariate regression model whereas WHR, WC, past history of hypertension, diabetes as well as hypercholesterolemia (all with *p* <0.1 in univariate analysis) were not entered into the multivariate model. The second multivariate model was further adjusted for energy-adjusted dietary intakes of fiber, saturated fat, carbohydrates and protein as these nutrients were previously reported to be associated with NAFLD [32]. Similarly, the multicollinearity problem was checked and no significant multicollinearity problem was detected. Furthermore, we examined whether the association between estimated NEAP or PRAL and the presence of NAFLD varied according to sex and BMI (normal weight group <23 vs. overweight and obese group ≥23 kg/m^2^). Interaction between sex or BMI status and estimated NEAP or PRAL was tested by addition of cross-product terms to the multivariate models. Interactions were not significant, thus all analyses are presented in the total population. To further check the possibility of reverse causation, a sensitivity analysis excluding 206 participants with medical history of hypertension, diabetes, hypercholesterolemia and ischemic heart disease was performed (n = 587). All models were adjusted for the same variables mentioned above.

Since NAFLD is benign in most of the cases, we further examined the association of the degree of liver fibrosis with estimated NEAP or PRAL. Binary logistic regression models were applied to assess both the unadjusted and the adjusted associations between estimated NEAP or PRAL and the presence of possible advanced fibrosis. Linear regression models were also used to examine both the unadjusted and the adjusted associations of estimated NEAP or PRAL with liver stiffness (as continuous variable). All models were adjusted for the same variables mentioned above. An α level of 5% was used as the level of significance.

## Results

There was no major difference in the baseline characteristics between the included subjects and the excluded subjects (data not shown). The mean ± SD age of 793 subjects was 48.1 ± 10.6 years (range 19–72 years), and 330 (41.6%) were male. All subjects were ethnic Chinese. 220 subjects were diagnosed with NAFLD and the prevalence of NAFLD was 27.7%. Subjects with NAFLD were older, and were likely to be male and current drinkers than subjects without NAFLD (all *p*<0.05). They also had significantly higher BMI, higher WC, higher WHR, and higher percentage of hypertension, diabetes, hypercholesterolemia, and metabolic syndrome than those without NAFLD (Table 1). Subjects with NAFLD tended to have a higher estimated NEAP or PRAL (Table 2). Subjects with NAFLD and those without NAFLD showed similar nutrient intakes and food group intakes except for the intakes of milk and milk products, and meat, poultry and organ meat.

**Table 1:** Baseline characteristics between subjects with and without NAFLD (n = 793).

Variables	No NAFLD (n = 573)		NAFLD (n = 220)		*P* ^1^
	Mean / n	SD / %	Mean / n	SD / %	
Age (years)	47.0	10.8	51.9	9.3	<0.001
BMI (kg/m^2^)	21.7	3.0	25.5	3.5	<0.001
WHR	0.84	0.08	0.90	0.06	<0.001
WC (cm)	78.3	9.2	89.2	7.6	<0.001
Male (%)		37.0		53.6	<0.001
Current smoker (%)		8.4		8.6	0.906
Current drinker (%)		16.6		25.0	0.007
Hypertension (%)		9.4		31.8	<0.001
Diabetes (%)		2.6		8.2	<0.001
Hypercholesterolemia (%)		10.2		16.4	0.016
Metabolic syndrome (%)		7.9		47.7	<0.001
ALT (IU/L)^2^	19	15–25	31	23–42	<0.001
AST (IU/L)^2^	19	17–22	22	18–27	<0.001
Intraheptic triglyceride content (%)^2^	1.2	0.7–2.4	9.8	7.0–15.4	<0.001
Liver stiffness measurement (kPa)^2^ ^,^ ^3^	4.1	3.5–4.9	4.6	4.0–5.7	<0.001
Liver stiffness (> = 7.9 kPa)		3.8		7.7	0.039

^1^ Group difference by independent t test or non-parametric Mann-Whitney U test for continuous variables and chi square or Fisher’s exact test for categorical variables

^2^ Median and interquartile range

^3^ Included 654 subjects (182 with fatty liver and 472 without fatty liver) with valid transient elastography examination.

**Table 2:** Dietary acid load and intakes of selected nutrients and food groups between subjects with and without NAFLD (n = 793).

Dietary variables	No NAFLD (n = 573)		NAFLD (n = 220)		*P* ^1^
	Mean	SD	Mean	SD	
NEAP (mEq/d)					
Raw	75.6	20.6	78.5	22.6	0.080
Energy adjusted	75.2	20.5	77.9	22.5	0.101
PRAL (mEq/d)					
Raw	23.0	16.5	25.3	17.9	0.088
Energy adjusted	23.6	14.2	25.7	16.1	0.072
Energy intake (kcal/d)	2066.1	610.2	2144.6	665.7	0.114
% energy from carbohydrates	50.5	8.0	49.7	8.2	0.211
% energy from total fat	32.6	7.1	32.5	7.1	0.834
% energy from protein	17.0	3.4	17.4	3.5	0.171
Carbohydrates (g/d)	260.6	89.6	267.1	99.2	0.375
Total fat (g/d)	74.7	27.2	77.2	27.8	0.242
Saturated fat (g/d)	17.1	7.0	17.6	7.2	0.388
Cholesterol (mg/d)	289.3	133.6	305.0	138.6	0.142
Protein (g/d)	88.0	32.8	92.5	33.5	0.085
Fiber (g/d)^2^	13.8	10.5–18.3	14.2	9.8–18.4	0.778
Potassium (mg/d)^2^	2079.4	1652.4–2691.1	2134.4	1669.3–2736.3	0.627
Vitamin C (mg/d)^2^	105.5	69.6–160.8	103.0	62.9–161.3	0.282
Calcium (mg/d)^2^	508.4	397.6–681.6	530.6	389.6–693.1	0.781
Magnesium (mg/d)	280.2	101.2	292.2	111.6	0.148
Phosphorus (mg/d)	1111.7	386.5	1161.9	414.3	0.109
Grains and cereals (g/d)^2^	521.4	395.7–685.7	556.4	414.3–740.0	0.079
Fruits and dried fruits (g/d)^2^	175.0	103.6–272.9	165.5	100.0–271.1	0.633
Vegetables and legumes (g/d)^2^	161.4	100.8–251.1	144.1	99.4–234.3	0.242
Soy and soy products (g/d)^2^	34.3	3.6–75.3	35.7	7.1–72.4	0.793
Egg and egg products (g/d)^2^	14.3	7.1–24.5	14.3	5.0–28.6	0.870
Meat, poultry and organ meats (g/d)^2^	114.3	77.1–170.3	130.0	79.0–178.3	0.045
Fish and seafood (g/d)^2^	60.7	33.6–98.6	64.4	34.0–106.4	0.497
Milk and milk products (g/d)^2^	14.3	0.9–49.1	8.6	0.0–33.2	0.013

^1^ Group difference by independent t test or non-parametric Mann-Whitney U test

^2^ Median and interquartile range.

Estimated NEAP was positively associated with total protein intake, and was negatively associated with the intakes of vitamin C, fiber, and various alkaline producing minerals, such as magnesium and potassium (*p*<0.05, Table 3). With respect to specific food groups, higher estimated NEAP was associated with higher consumption of protein rich animal foods, and lower consumption of fruits, soy and soy products, and vegetables and legumes (*p*<0.05, Table 3). Similar correlations were observed when the analyses were performed using PRAL (Table 3).

**Table 3:** Correlation between estimated NEAP or PRAL and selected nutrients and food groups (n = 793).

Energy adjusted nutrients/food groups	Energy adjusted estimated NEAP (mEq/d)		Energy adjusted estimated PRAL (mEq/d)	
	r	*P*	r	*P*
Nutrients				
Total protein (g)	0.28	<0.001	0.57	<0.001
Vitamin C (mg)^1^	-0.59	<0.001	-0.46	<0.001
Calcium (mg)^1^	-0.45	<0.001	-0.27	<0.001
Phosphorus (mg)	-0.09	0.010	0.25	<0.001
Fiber (g)^1^	-0.67	<0.001	-0.51	<0.001
Magnesium (mg)	-0.46	<0.001	-0.26	<0.001
Potassium (mg)^1^	-0.65	<0.001	-0.34	<0.001
Food groups (g)				
Grains and cereals^1^	0.01	0.835	0.05	0.134
Egg and egg products^1^	0.15	<0.001	0.18	<0.001
Fish and seafood^1^	-0.04	0.254	0.12	<0.001
Fruits and dried fruits^1^	-0.66	<0.001	-0.54	<0.001
Meat, poultry and organ meats^1^	0.44	<0.001	0.54	<0.001
Milk and milk products^1^	-0.09	0.011	-0.05	0.203
Soy and soy products^1^	-0.13	<0.001	-0.07	0.053
Vegetables and legumes^1^	-0.46	<0.001	-0.33	<0.001

^1^ By Spearman’s rank correlation.

Table 4 shows the results of logistic regression models linking energy adjusted estimated NEAP or PRAL and the likelihood of having NAFLD. The multivariate model adjusting for age, sex, BMI, current drinker status and the presence of metabolic syndrome suggested that a higher estimated NEAP was associated with an increased likelihood of having NAFLD. Further adjustment for dietary intakes of fiber, saturated fat, carbohydrates and protein did not change the results. Sensitivity analyses excluding participants with selected chronic diseases that could have affected their diets showed similar results (data not shown). However, such significant association was not observed when the regression analyses were performed using PRAL. Regarding the association between dietary acid load and the presence of possible advanced fibrosis, no significant association was detected in both unadjusted and adjusted models, regardless of markers for dietary acid load used. Analyses performed with liver stiffness as a continuous variable did not change the results (data not shown).

**Table 4:** Logistic regression linking NEAP or PRAL and the presence of NAFLD (n = 793).

Models	Energy adjusted estimated NEAP (mEq/d)			Energy adjusted estimated PRAL (mEq/d)		
	OR	95% CI	*P*	OR	95% CI	*P*
Unadjusted (per 20 unit increase)	1.13	0.96–1.32	0.102	1.22	1.00–1.49	0.073
Multivariate adjusted^1^						
NEAP (per 20 unit increase)	1.25	1.02–1.52	0.022	—-		
PRAL (per 20 unit increase)	—-			1.15	0.87–1.51	0.341
Sex	1.64	1.08–2.48	0.020	1.66	1.08–2.54	0.020
Age (per 10 years increase)	1.31	1.06–1.63	0.015	1.25	1.00–1.55	0.045
BMI (kg/m^2^)	1.35	1.28–1.45	<0.001	1.35	1.27–1.44	<0.001
Current drinker	0.95	0.57–1.58	0.835	0.92	0.56–1.53	0.758
Metabolic syndrome	5.14	3.24–8.15	<0.001	5.12	3.23–8.12	<0.001
Multivariate adjusted^2^						
NEAP (per 20 unit increase)	1.32	1.01–1.74	0.049	—-		
PRAL (per 20 unit increase)	—-			1.13	0.68–1.88	0.627
Sex	2.47	1.08–5.64	0.032	2.62	1.15–5.98	0.022
Age (per 10 years increase)	1.31	1.04–1.66	0.018	1.27	1.02–1.58	0.033
BMI (kg/m^2^)	1.36	1.27–1.45	<0.001	1.35	1.27–1.45	<0.001
Current drinker	0.92	0.54–1.55	0.743	0.86	0.51–1.46	0.577
Metabolic syndrome	5.19	3.26–8.25	<0.001	5.23	3.29–8.31	<0.001
Energy adjusted fiber intake (g/d)	1.01	0.96–1.07	0.734	0.99	0.93–1.05	0.640
Energy adjusted saturated fat intake (g/d)	0.98	0.92–1.04	0.424	0.97	0.91–1.03	0.314
Energy adjusted carbohydrates intake (g/d)	1.00	0.99–1.00	0.385	1.00	0.99–1.00	0.337
Energy adjusted protein intake (g/d)	0.99	0.98–1.00	0.175	0.99	0.98–1.01	0.406

^1^ Variables with *p*<0.1 in the univariate analysis were entered into the multivariate model

^2^ Further adjustment for energy-adjusted dietary intakes of fiber, saturated fat, carbohydrates and protein as these nutrients were previously reported to be associated with NAFLD [32].

## Discussion

Our data showed that higher estimated NEAP but not PRAL was associated with an increased likelihood of having NAFLD in Hong Kong Chinese adults, whereas both markers of dietary acid load were not associated with the presence of possible advance fibrosis.

To our knowledge, only one study has examined the association of dietary acid-base load and risk of NAFLD in a group of healthy Caucasian young men and women aged 18–25 years, and the study showed that higher dietary acidity as measured using the PRAL in adolescence may be prospectively associated with hepatic lipid accumulation in females [14]. However, one of the major limitations of this prospective study by Krupp *et al*. was the use of ALT, HSI, and FLI as surrogates of NAFLD. In our study, we used ^1^H-MRS, one of the most accurate non-invasive tests for measuring hepatic steatosis, and included data on fibrosis collected using transient elastography.

The potential link between estimated NEAP and NAFLD remains unclear. Accumulating evidence shows that reduced levels of GH and IGF-I in adults is associated with an increased prevalence of NAFLD and progression to NASH or liver cirrhosis [7–9]. Substantial evidence from animal studies and human studies also shows that metabolic acidosis suppresses GH secretion and IGF-I response [10, 11, 33]. In humans, Wiederkehr and colleagues reported that correction of renal acidosis by oral citrate administration to haemodialysis patients partly corrected IGF-1 levels and reversed GH insensitivity [11]. Therefore, it is postulated that high estimated NEAP may contribute to hepatic triglyceride accumulation possibly through its influence on the GH-IGF-I system. In contrast to the prospective study by Krupp’s *et al*., we showed that higher estimated NEAP but not PRAL was modestly associated with NAFLD prevalence. Data on the association of estimated NEAP with NAFLD were not reported in Krupp’s study, thus the association between different measures of dietary acid load and NAFLD remains uncertain. Differences in the outcome measures, the age range, as well as the dietary practice of the studied samples between our study and Krupp’s study make the comparison of findings difficult. More studies are therefore needed to ascertain the link between dietary acid load and NAFLD.

Epidemiological data on the association between diet or nutrient composition and the development of NAFLD are limited. Previous studies on this aspect were mainly conducted in Caucasian populations and were seldom investigated among Chinese population. Existing evidence generally supports that a diet high in energy, simple carbohydrates, saturated fat, and cholesterol intakes but low in fiber content increases the risk of NAFLD [34, 35]. The relationship between protein intake and NAFLD risk is more complex. Dietary protein is part of the estimated NEAP or the PRAL and increasing protein intake increases dietary acidity. Therefore, a high protein intake could contribute to a GH resistance in the liver, through its influence on the acid-base balance. In contrast, an elevated IGF-I concentration has been reported in subjects with type 2 diabetes following a high-protein diet [36]. Experiments in rodents also showed that a high-protein and low-carbohydrate diet improved carbohydrate metabolism and decreased hepatic steatosis by inhibition of de novo lipogenesis [37, 38]. Therefore, we tried to further adjust for dietary fiber, saturated fat, carbohydrates and protein in our multivariate analysis and the association between estimated NEAP and the likelihood of prevalent NAFLD was slightly attenuated but remained significant, possibly suggesting that diet-induced acid load may constitute a nutritional factor with influence on NAFLD development. However, our findings were in line with other previous studies that metabolic syndrome is still the strongest factor associated with NAFLD [15, 39]. Therefore, the magnitude of effect of diet on NAFLD remains to be examined.

The strengths of our study include relatively large sample size, recruitment of subjects from the general population, and the use of ^1^H-MRS and transient elastography to assess hepatic steatosis and fibrosis respectively. However, our study has several limitations. First, our study was a cross-sectional study, thus the causal relationship between estimated NEAP and the likelihood of NAFLD cannot be determined. We tried to conduct a sensitivity analysis excluding participants with chronic diseases that could have affected their diets and similar results were observed. These results suggest that reverse causation seems an unlikely explanation for our findings. Second, the validated FFQ used in this study captured only the short term dietary and food intakes (i.e. the past week) of the participants. Perhaps longer-term dietary and food intakes are more valuable in examining the relationship between diet and risk of chronic diseases. Third, we did not have data on GH and IGF-I as well as biochemical markers regarding acid base load, such as serum anion gap and bicarbonate in the study, thus no evidence of the presence of low grade/mid metabolic acidosis in the subjects with NAFLD is available. The association of estimated NEAP as the underlying mechanism with NAFLD prevalence should therefore be regarded as speculation rather than conviction. Forth, the number of cases with possible advanced fibrosis may be insufficient to allow for an examination of the association between diet-induced acid load and the presence of possible advanced fibrosis. Besides, although we controlled for various common factors and major medical conditions in the analysis, residual potential confounding from some other important factors related to the development of NAFLD, such as physical activity level might still be present.

In conclusion, our findings suggest that there may be a modest association between diet-induced acid load and NAFLD. More studies are needed to ascertain the link between diet-induced acid load and NAFLD and to investigate the underlying mechanisms.

## Supporting Information

S1 File Food Frequency Questionnaire used in the study. (XLS)

## References

[1] Amarapurkar DN, Hashimoto E, Lesmana LA, Sollano JD, Chen PJ, Goh KL. How common is non-alcoholic fatty liver disease in the Asia-Pacific region and are there local differences? J Gastroenterol Hepatol. 2007;22:788–793. 17565631 10.1111/j.1440-1746.2007.05042.x

[2] Chitturi S, Wong VW, Farrell G. Nonalcoholic fatty liver in Asia: Firmly entrenched and rapidly gaining ground. J Gastroenterol Hepatol. 2011;26:S163–S172. 10.1111/j.1440-1746.2010.06548.x 21199528

[3] Caldwell SH, Oelsner DH, Iezzoni JC, Hespenheide EE, Battle EH, Driscoll CJ. Cryptogenic cirrhosis: clinical characterization and risk factors for underlying disease. Hepatology. 1999;29:664–669. 10051466 10.1002/hep.510290347

[4] Farrell GC, Larter CZ. Nonalcoholic fatty liver disease: from steatosis to cirrhosis. Hepatology. 2006;43:S99–S112. 16447287 10.1002/hep.20973

[5] Hamaguchi M, Kojima T, Takeda N, Nagata C, Takeda J, Sarui H, et al Nonalcoholic fatty liver disease is a novel predictor of cardiovascular disease. World J Gastroenterol. 2007;13:1579–1584. 17461452 10.3748/wjg.v13.i10.1579 PMC4146902

[6] Wong VW, Wong GL, Yip GW, Lo AO, Limquiaco J, Chu WC, et al Coronary artery disease and cardiovascular outcomes in patients with non-alcoholic fatty liver disease. Gut. 2011;60:1721–1727. 10.1136/gut.2011.242016 21602530

[7] Ichikawa T, Nakao K, Hamasaki K, Furukawa R, Tsuruta S, Ueda Y, et al Role of growth hormone, insulin-like growth factor 1 and insulin-like growth factor-binding protein 3 in development of non-alcoholic fatty liver disease. Hepatol Int. 2007;1:287–294. 10.1007/s12072-007-9007-4 19669352 PMC2716823

[8] Takahashi Y. Essential roles of growth hormone (GH) and insulin-like growth factor-I (IGF-I) in the liver. Endocrine. 2012;59:955–962. 22986486 10.1507/endocrj.ej12-0322

[9] Xu L, Xu C, Yu C, Miao M, Zhang X, Zhu Z, et al Association between serum growth hormone levels and nonalcoholic fatty liver disease: a cross-sectional study. PLoS ONE. 2012;7:e44136 10.1371/journal.pone.0044136 22952901 PMC3432067

[10] Mitch WE. Metabolic and clinical consequences of metabolic acidosis. J Nephrol. 2006;19:S70–75. 16736444

[11] Wiederkehr MR, Kalogiros J, Krapf R. Correction of metabolic acidosis improves thyroid and growth hormone axes in haemodialysis patients. Nephrol Dial Transplant. 2004;19:1190–1197. 14993483 10.1093/ndt/gfh096

[12] Remer T. Influence of diet on acid-base balance. Semin Dial. 2000;13:221–226. 10923348 10.1046/j.1525-139x.2000.00062.x

[13] Pizzorno J, Frassetto LA, Katzinger J. Diet-induced acidosis: is it real and clinically relevant? Br J Nutr. 2010;103:1185–1194. 10.1017/S0007114509993047 20003625

[14] Krupp D, Johner SA, Kalhoff H, Buyken AE, Remer T. Long-term dietary potential renal acid load during adolescence is prospectively associated with indices of nonalcoholic fatty liver disease in young women. J Nutr. 2012;142:313–319. 10.3945/jn.111.150540 22223573

[15] Wong VW, Chu WC, Wong GL, Chan RS, Chim AM, Ong A, et al Prevalence of non-alcoholic fatty liver disease and advanced fibrosis in Hong Kong Chinese: a population study using proton-magnetic resonance spectroscopy and transient elastography. Gut. 2012;61:409–415. 10.1136/gutjnl-2011-300342 21846782

[16] Wong VW, Wong GL, Chu WC, Chim AM, Ong A, Yeung DK, et al Hepatitis B virus infection and fatty liver in the general population. J Hepatol. 2012;56:533–540. 10.1016/j.jhep.2011.09.013 22027575

[17] Farrell GC, Chitturi S, Lau GK, Sallano JD, Asia-Pacific Working Party on NAFLD. Guidelines for the assessment and management of non-alcoholic fatty liver disease in the Asia-Pacific region: executive summary. J Gastroenterol Hepatol. 2007; 22:775–777. 17565629 10.1111/j.1440-1746.2007.05002.x

[18] Alberti KG, Eckel RH, Grundy SM, Zimmet PZ, Cleeman JI, Donato KA, et al Harmonizing the metabolic syndrome: a joint interim statement of the International Diabetes Federation Task Force on Epidemiology and Prevention; National Heart, Lung, and Blood Institute; American Heart Association; World Heart Federation; International Atherosclerosis Society; and International Association for the Study of Obesity. Circulation. 2009;120:1640–1645. 10.1161/CIRCULATIONAHA.109.192644 19805654

[19] Wong VW, Wong GL, Tsang SW, Fan T, Chu WC, Woo J, et al High prevalence of colorectal neoplasm in patients with non-alcoholic steatohepatitis. Gut. 2011;60:829–836. 10.1136/gut.2011.237974 21339204

[20] Browning JD, Szczepaniak LS, Dobbins R, Nuremberg P, Horton JD, Cohen JC, et al Prevalence of hepatic steatosis in an urban population in the United States: impact of ethnicity. Hepatology. 2004;40:1387–1395. 15565570 10.1002/hep.20466

[21] Wong VW, Vergniol J, Wong GL, Foucher J, Chan HL, Le Bail B, et al Diagnosis of fibrosis and cirrhosis using liver stiffness measurement in nonalcoholic fatty liver disease. Hepatology. 2010;51:454–462. 10.1002/hep.23312 20101745

[22] Woo J, Leung SSF, Ho SC, Lam TH, Janus ED. A food frequency questionnaire for use in the Chinese population in Hong Kong: Description and examination of validity. Nutr Res. 1997;17:1633–1641.

[23] Yang Y, Wang G, Pan X. China Food Composition 2002. 2002 ed. Peking, China: University Medical Press; 2002.

[24] Yang Y, Wang G, Pan X. China Food Composition 2004. 2002 ed. Peking, China: University Medical Press; 2004.

[25] Centre for Food Safety. Nutrient Information Inquiry. Hong Kong SAR: Centre for Food Safety; 2006.

[26] Willett WC, Howe GR, Kushi LH. Adjustment for total energy intake in epidemiologic studies. Am J Clin Nutr. 1997;65:1220S–1228S. 9094926 10.1093/ajcn/65.4.1220S

[27] Frassetto LA, Lanham-New SA, Macdonald HM, Remer T, Sebastian A, Tucker KL, et al Standardizing terminology for estimating the diet-dependent net acid load to the metabolic system. J Nutr. 2007;137:1491–1492. 17513412 10.1093/jn/137.6.1491

[28] Frassetto LA, Todd KM, Morris RC Jr, Sebastian A. Estimation of net endogenous noncarbonic acid production in humans from diet potassium and protein contents. Am J Clin Nutr. 1998;68:576–583. 9734733 10.1093/ajcn/68.3.576

[29] Remer T, Dimitriou T, Manz F. Dietary potential renal acid load and renal net acid excretion in healthy, free-living children and adolescents. Am J Clin Nutr. 2003;77:1255–1260. 12716680 10.1093/ajcn/77.5.1255

[30] Frassetto LA, Morris RC Jr, Sebastian A. A practical approach to the balance between acid production and renal acid excretion in humans. J Nephrol. 2006;19:S33–S40. 16736439

[31] Chan RS, Woo J, Chan DC, Cheung CS, Lo DH. Estimated net endogenous acid production and intake of bone health-related nutrients in Hong Kong Chinese adolescents. Eur J Clin Nutr. 2009;63:505–512. 10.1038/ejcn.2008.3 18231119

[32] Kargulewicz A, Stankowiak-Kulpa H, Grzymisławski M. Dietary recommendations for patients with nonalcoholic fatty liver disease. Prz Gastroenterol. 2014;9:18–23. 10.5114/pg.2014.40845 24868294 PMC4027841

[33] Challa A, Krieg RJJ, Thabet MA, Veldhuis JD, Chan JC. Metabolic acidosis inhibits growth hormone secretion in rats: mechanism of growth retardation. Am J Physiol. 1993;265:E547–E553. 8238328 10.1152/ajpendo.1993.265.4.E547

[34] McCarthy EM, Rinella ME. The Role of Diet and Nutrient Composition in Nonalcoholic Fatty Liver Disease. J Acad Nutr Diet. 2012;112:401–409. 10.1016/j.jada.2011.10.007 22717200

[35] Mouzaki M, Allard JP. The role of nutrients in the development, progression, and treatment of nonalcoholic fatty liver disease. J Clin Gastroenterol. 2012;46:457–467. 10.1097/MCG.0b013e31824cf51e 22469640

[36] Gannon MC, Nuttall FQ. Effect of a high-protein diet on ghrelin, growth hormone, and insulin-like growth factor-I and binding proteins 1 and 3 in subjects with type 2 diabetes mellitus. Metabolism. 2011;60:1300–1311. 10.1016/j.metabol.2011.01.016 21406307

[37] Blouet C, Mariotti F, Azzout-Marniche D, Bos C, Mathe V, Tome D, et al The reduced energy intake of rats fed a high-protein low-carbohydrate diet explains the lower fat deposition, but macronutrient substitution accounts for the improved glycemic control. J Nutr. 2006;136:1849–1854. 16772448 10.1093/jn/136.7.1849

[38] Pichon L, Huneau JF, Fromentin G, Tome D. A high-protein, high-fat, carbohydrate-free diet reduces energy intake, hepatic lipogenesis, and adiposity in rats. J Nutr. 2006;136:1256–1260. 16614413 10.1093/jn/136.5.1256

[39] Wong VW, Hui AY, Tsang SW, Chan JL, Tse AM, Chan KF, et al Metabolic and adipokine profile of Chinese patients with nonalcoholic fatty liver disease. Clin Gastroenterol Hepatol. 2006;4:1154–1161. 16904946 10.1016/j.cgh.2006.06.011

